# Supplementary material for: Comparative Cyto-Histological Genetic Profile in a Series of Differentiated Thyroid Carcinomas
Source: Diagnostics (Basel). 2024 Jan 27;14(3):278. doi: 10.3390/diagnostics14030278 (PMC10855767; doi:10.3390/diagnostics14030278)
Supplement: Supplementary file 1 [file diagnostics-14-00278-s001.zip › Supplementary Tables Diagnostics v2.pdf]

Supplementary Table S1. Mutational frequency through Bethesda categories.

	Bethesda categories						
Mutations	ND n=15	B n=47	AUS/FLUS n= 43	FT/SFN n=58	SM n=42	M n=54	Total
TERTp	0	0	1	1	0	0	2
BRAF	0	0	1	4	8	30	43
TERTp+BRAF	0	0	1	1	0	4	6
RAS (NRAS,HRAS, KRAS)	0	2	5	8	7	4	26
TERTp+RAS	0	0	0	1	0	0	1
Total	0	2	8	15	15	38	78

Legend: ND: Non-diagnostic, B: Benign, AUS: Atypia of Undetermined Significance, FN: Follicular Neoplasm, SM: Suspicious for malignancy and M: Malignant. *TERTp* - telomerase reverse transcriptase promoter; *BRAF* - B-Raf proto-oncogene, serine/threonine kinase; *NRAS* - NRAS proto-oncogene, GTPase; *HRAS* - HRas proto-oncogene, GTPase, and *KRAS* - KRAS proto-oncogene, GTPase; RAS- RAS proto-oncogene, GTPase.

Supplementary Table S2. Mutation types in cytology and histology of indeterminate nodules.

Genetic mutations	Cytology (mutated)<br>n=25 (24.8%)				Histology (mutated)<br>n=48 (47.5%)			
Histology of Indeterminate nodules	n=	Benign<br>n=1	Malignant<br>n=24	Mutation type	n=	Benign<br>n=2	Malignant<br>n=46	Mutation type
TERTp	94	0 (0%)	4 (5.6%)		99	0 (0%)	11 (14.5%)	
AUS		0	2	1 (-124 G>A)<br>1 (-146 G>A)		0	5	4 (-124 G>A)<br>1 (-146G>A)
FN		0	2	2 (-124 G>A)		0	6	4 (-124 G>A)<br>2 (-146 G>A)
BRAF	97	0 (0%)	7 (9.3%)		100	0 (0%)	13 (16.9%)	
AUS		0	1	1 (p.V600E)		0	5	5 (p.V600E)
FN		0	6	6 (p.V600E)		0	8	8 (p.V600E)
RAS	97	1 (1%)	13(17.3%)		98	2 (8.7%)	22 (29.3%)	
AUS		1	4	1 B (p.Q61K)<br>4 M (p.Q61R)		2	10	2 B (p.Q61K/p.G12A)<br>8 M (p.Q61R)
FN		0	9	7 M (p.Q61R)<br>2 M (p.Q61K)		0	12	2 M(p.Q61K/p.G12R)<br>9 M (p.Q61R)<br>3 M (p.Q61K)

Legend: AUS: Atypia of Undetermined Significance, FN: Follicular Neoplasm. *TERTp* - telomerase reverse transcriptase promoter; *BRAF* - B-Raf proto-oncogene, serine/threonine kinase; RAS- RAS proto-oncogene, GTPase.

Supplementary Table S3. Clinicopathologic features present in the PTC variants.

Clinicopathologic features	PTC variants n=180							
	c-PTC n=73 (40.5%)	FV-PTC n=73 (40.5%)	EV-PTC n=5 (2.8%)	OV-PTC n=15 (8.3%)	SV-PTC n= 10 (5.6%)	TC-PTC n=3 (1.7%)	CV-PTC n=1 (0.6%)	TOTAL n= 180 (100%)
Extrathyroidal invasion	36 (49.3%)	10 (13.7%)	0 (0%)	5 (33.3%)	2 (20%)	2 (66.7%)	1 (100%)	56 (31.1%)
Capsule invasion	37 (50.7%)	41 (56.2%)	1 (20%)	12 (80%)	2 (20%)	3 (100%)	1 (100%)	97 (53.9%)
Vascular invasion	18 (24.7%)	12 (16.4%)	3 (60%)	5 (33.3%)	0 (0%)	2 (66.7%)	1 (100%)	41 (22.8%)
Lymphatic invasion	19 (26%)	10 (13.7%)	1 (20%)	5 (33.3%)	0 (0%)	1 (33.3%)	0 (0%)	36 (20%)
Fibrosis	51 (69.9%)	35 (48%)	2 (40%)	13 (86.7%)	5 (50%)	2 (66.7%)	1 (100%)	109 (60.6%)
Inflammatory infiltrate	43 (58.9%)	18 (24.7%)	0 (0%)	7 (46.7%)	1 (10%)	3 (100%)	0 (0%)	72 (40%)
Tall cell								
<30%	17 (23.3%)	1 (1.4%)	0 (0%)	4 (26.7%)	0 (0%)	0 (0%)	0 (0%)	22 (12.2%)
≥30%	0 (0%)	0 (0%)	0 (0%)	0 (0%)	0 (0%)	3 (100%)	0 (0%)	3 (1.7%)
Oncocytic component								
<50%	21(28.8%)	7 (9.6%)	0 (0%)	0 (0%)	0 (0%)	2 (66.7%)	0 (0%)	30 (16.7%)
≥50%	5 (6.8%)	3 (4.1%)	0 (0%)	15 (100%)	1 (10%)	1 (33.3%)	0 (0%)	25 (13.9%)
Psammoma bodies	17 (23.3%)	3 (4.1%)	1 (20%)	2 (13.3%)	0 (0%)	2 (66.7%)	0 (0%)	25 (13.9%)
Calcification	18 (24.7%)	10 (13.7%)	1 (20%)	3 (20%)	0 (0%)	2 (66.7%)	0 (0%)	34 (18.9%)
Necrosis	2 (2.7%)	1 (1.4%)	0 (0%)	2 (13.3%)	0 (0%)	0 (0%)	0 (0%)	5 (2.8%)
Focality								
unifocal	43(58.9%)	44 (60.3%)	5(100%)	9 (60%)	6 (60%)	3 (100%)	1 (100%)	111 (61.7%)
multifocal	30 (41.1%)	29 (39.7%)	0 (0%)	6 (40%)	4 (40%)	0 (0%)	0 (0%)	69 (38.3%)
Laterality								
unilateral	50 (68.5%)	51 (69.9%)	5(100%)	7(46.7%)	6 (60%)	3 (100%)	0 (0%)	122 (67.8%)
bilateral	23 (31.5%)	22 (30.1%)	0 (0%)	8 (53.3%)	4 (40%)	0 (0%)	1 (100%)	58 (32.2%)
Lymph node metastasis	25 (34.2%)	7 (9.6%)	0 (0%)	5(33.3%)	0 (0%)	1 (33.3%)	0 (0%)	38 (21.1%)
Distant metastasis	4 (9.9%)	4 (2.8%)	0 (0%)	0 (0%)	0 (0%)	0 (0%)	1 (100%)	9 (5%)

Legend : PTC– papillary thyroid carcinoma; PTC variants: c-PTC- classical; FV-PTC -follicular; EV-PTC – encapsulate; SV- solid; TC-PTC- tall cells; OV-PTC- oncocytic ; CV-PTC- columnar.

Supplementary Table S4. Mutational status of cytology and histology samples within PTC variants.

Mutations	PTC variants							
	c-PTC n =74 (40.6%)	FV-PTC n= 74 (40.6%)	EV-PTC n=5 (2.7%)	OV-PTC n=15 (8.2%)	SV-PTC n= 10 (5.5%)	TC-PTC n=3 (1.7%)	CC-PTC n=1 (0.6%)	TOTAL n= 182 (100%)
TERTp								
Histology n=176	5	8	0	4	1	1	0	19 (10.8%)
Cytology n= 171	2	3	0	2	1	1	0	9 (5.3%)
BRAF								
Histology n=177	36	14	2	8	0	3	0	63 (35.6%)
Cytology n= 175	28	11	2	5	0	2	0	48 (27.4%)
RAS								
Histology n=176	7	22	1	5	3	0	0	38 (22.1%)
Cytology n= 171	7	13	1	3	1	0	0	25 (14.3%)
Total								
Histology	49	45	3	17	4	4	0	122
Cytology	38	27	3	10	2	3	0	83

Legend : PTC– papillary thyroid carcinoma; PTC variants: c-PTC- classical; FV-PTC -follicular; EV-PTC – encapsulate; SV- solid; TC-PTC- tall cells; OV-PTC- oncocytic ; CV-PTC- columnar. *TERTp* - telomerase reverse transcriptase promoter; *BRAF* - B-Raf proto-oncogene, serine/threonine kinase;; *RAS*- RAS proto-oncogene, GTPase.

Supplementary Table S5A. Associations between *TERTp* mutation and clinicopathological features in Papillary Thyroid Carcinomas.

Clinicopathological characteristics n (%)	Wild-Type n (%)	Mutated n (%)	p-value
Vascular invasion n=226			
Absent 186 (82.3)	175 (84.5)	11 (57.9)	0.004
Present 40 (17.7)	32 (15.5)	8 (42.0)	
Oncocytic component n=224			
Absent 172 (76.8)	162 (79.0)	10 (52.6)	0.017
Present 52 (23.2)	43 (21.1)	9 (47.4)	

Supplementary Table S5B. Associations between *BRAF* mutation and clinicopathological features in Papillary Thyroid Carcinomas.

Clinicopathological characteristics n (%)	Wild-Type n (%)	Mutated n (%)	p-value
Mean tumor size (SD) (mm) (n=227)	30.76 (14.31)	21.65 (14.30)	<0.001
Extra thyroidal invasion n=227			
Absent 172 (82.3)	140 (85.4)	32 (50.8)	<0.001
Present 55 (17.7)	24 (14.6)	31 (49.2)	
Capsule invasion n=227			
Absent 132 (58.1)	112 (68.3)	20 (31.7)	<0.001
Present 95 (41.9)	52 (31.7)	43 (68.3)	
Vascular invasion n=227			
Absent 187 (82.4)	149 (90.9)	38 (60.3)	<0.001
Present 40 (17.6)	15 (9.1)	25 (39.7)	
Lymphatic invasion n=227			
Absent 192 (84.6)	151 (92.1)	41 (65.1)	<0.001
Present 35 (15.4)	13 (7.9)	22 (34.9)	
Fibrosis n=227			
Absent 120 (52.9)	102 (62.2)	18 (2.6)	<0.001
Present 107 (47.1)	62 (37.8)	45 (71.4)	
Inflammatory infiltrate n=227			
Absent 154 (67.8)	129 (78.7)	25 (39.7)	<0.001
Present 73 (32.2)	35 (21.3)	38 (60.3)	
Tall cells n=227			
Absent 203 (89.4)	157 (95.7)	46 (73)	<0.001
Present 24 (10.6)	7 (4.3)	17 (27)	

Oncocytic component n=225			
Absent 173 (76.9)<br>Present 52 (23.1)	134 (82.2)<br>29 (17.8)	39 (62.9)<br>23 (27.1)	0.002
Psammoma bodies n=227			
Absent 203 (89.4)<br>Present 24 (10.6)	157 (95.7)<br>7 (4.3)	46 (73)<br>17 (27)	<0.001
Calcifications n=227			
Absent 194 (85.5)<br>Present 33 (14.5)	150 (91.5)<br>14 (8.5)	44 (69.8)<br>19 (30.2)	<0.001
Focality n=226			
Unifocal 118 (52.2)<br>< 5 focos 65 (28.8)<br>>5 focos 43 (19)	85 (52.1)<br>40 (24.5)<br>38 (23.3)	33 (52.4)<br>25 (39.7)<br>5 (7.9)	<0.001
Lymph node metastases n=189			
Absent 152 (80.4)<br>Present 37 (19.6)	115 (91.3)<br>11 (8.7)	37 (58.7)<br>26 (41.3)	<0.001

Supplementary Table S5C. Associations between *NRAS* mutation and clinicopathological features in Papillary Thyroid Carcinomas.

Clinicopathological characteristics n (%)	Wild-Type n (%)	Mutated n (%)	p-value
Presence of capsule n=205			
Absent 98 (47.8)<br>Present 107 (52.2)	85 (51.5)<br>80 (48.5)	13 (32.5)<br>27 (67.5)	0.030
Focality n=221			
Unifocal 113 (51.1)<br><5 focos 65 (29.4)<br>>5 focos 43 (19.5)	89 (49.4)<br>50 (27.8)<br>41 (22.8)	24 (58.5)<br>15 (36.6)<br>2 (4.9)	0.030

Supplementary Table S6A. The discriminative ability of mutations for malignant diagnosis in Differentiated Thyroid Carcinomas (DTCs).

Mutations	DTCs (n=209) + Benign (n=50) n=259			
(n)	Se % (95%CI)	Sp % (95%CI)	PPV % (95%CI)	NPV % (95%CI)
TERTp				
Histology (254)	9.8 (6.1, 14.7)	100 (92.9, 100)	100 (83.2, 100)	21.4 (16.3, 27.2)
Cytology (246)	4.6 (2.1, 8.5)	100 (92.7, 100)	100 (66.4, 100)	20.7 (15.7, 26.4)
BRAF				
Histology (255)	31.2 (24.9, 38)	100 (92.9, 100)	100 (94.4, 100)	26.2 (20.1, 33)
Cytology (251)	24.3 (18.5, 30.8)	100 (92.7, 100)	100 (92.7, 100)	24.3 (18.5, 30.8)
RAS				
Histology (250)	21 (15.6, 27.3)	94 (83.5, 98.7)	93.3 (81.7, 98.6)	22.9 (17.4, 29.3)
Cytology (250)	12.9 (8.6, 18.4)	98 (89.1, 99.9)	96.3 (81, 99.9)	21.5 (16.3, 27.5)

TERTp - telomerase reverse transcriptase promoter; BRAF - B-Raf proto-oncogene, serine/threonine kinase; RAS- RAS proto-oncogene, GTPase.

Supplementary Table S6B. The discriminative ability of mutations for malignant diagnosis in Papillary Thyroid Carcinomas (PTCs).

Mutations	PTCs (n=180) + Benign (n=50) n=230			
(n)	Se % (95%CI)	Sp % (95%CI)	PPV % (95%CI)	NPV % (95%CI)
TERTp				
Histology (226)	10.8 (6.6, 16.3)	100 (92.9, 100)	100 (82.4, 100)	24.2 (18.5, 30.6)
Cytology (220)	5.26 (2.4, 9.8)	100 (92.7, 100)	100 (66.4, 100)	23.2 (17.7, 29.5)
BRAF				
Histology (227)	35.6 (28.6, 43.1)	100 (92.9, 100)	100 (94.3, 100)	30.5 (23.5, 38.1)
Cytology (224)	27.4 (21, 34.7)	100 (92.7, 100)	100 (92.6, 100)	27.8 (21.4, 35.1)
RAS				
Histology (222)	22.1 (16.1, 29)	94 (83.5, 98.7)	92.7 (80.1, 98.5)	26.0 (19.7, 33)
Cytology (224)	14.3 (9.5, 20.4)	98 (89.1, 99.9)	96.2 (80.4, 99.9)	24.2 (18.4, 30.8)

TERTp - telomerase reverse transcriptase promoter; *BRAF* - B-Raf proto-oncogene, serine/threonine kinase; RAS- RAS proto-oncogene, GTPase.

Supplementary Table S6C. The discriminative ability of mutations for malignant diagnosis in Indeterminate nodules.

Mutations	Indeterminate nodules (n=101) DTCs (n=78) + Benign (n=23)			
(n)	Se % (95%CI)	Sp % (95%CI)	PPV % (95%CI)	NPV % (95%CI)
TERTp				
Histology (99)	14.5 (7.45, 24.4)	100 (85.2, 100)	100 (71.5, 100)	26.1 (17.3, 36.6)
Cytology (94)	5.6 (1.53, 13.6)	100 (84.6, 100)	100 (39.8, 100)	24.4 (16, 34.6)

BRAF				
Histology (100)	16.9 (9.3, 27.1)	100 (85.2, 100)	100 (75.3, 100) 100	26.4 (17.7, 37)
Cytology (97)	9.3 (3.8, 18.3)	100 (84.6, 100)	(59, 100)	27.8 (21.4, 35.1)
RAS				
Histology (98)	29.3 (19.4, 41)	91.3 (72, 98.9)	91.7 (73, 99)	28.4 (18.5, 40.1)
Cytology (97)	17.3 (9.6, 27.8)	95.5 (77.2, 99.9)	92.9 (66, 99.8)	25.3 (16.4, 36)

Legend: *TERTp* - telomerase reverse transcriptase promoter; *BRAF* - B-Raf proto-oncogene, serine/threonine kinase; RAS- RAS proto-oncogene, GTPase.

. Se- Sensitivity; Sp- Specificity; PPV- Positive Predictive Value; NPV- Negative Predictive Value; CI- Confidence Interval.

Supplementary Table S7A. Profile of the molecular cyto-histological discordant cases in *TERTp* gene.

TERTp **Case number**	**Tumor size mm**	**Cytology by Bethesda categories**		**Histology**	
		**Diagnosis**	**Molecular status**	**Diagnosis**	**Molecular status**
69/70	25	II	WT	FV-PTC	-124 (G>A)
147/148	28	II	WT	c-PTC	-124 (G>A)
153/154	50	VI	-124 (G>A)	FV-PTC	WT
187/188	35	VI	WT	c-PTC	-146 (G>A)
197/198	15	III	WT	FV-PTC	-146 (G>A)
205/206	15	I	WT	c-PTC	-146 (G>A)
223/224	15	IV	WT	FV-PTC	-146 (G>A)
249/250	15	IV	WT	c-PTC	-146 (G>A)
259/260	28	IV	WT	FV-PTC	-124 (G>A)
261/262	40	I	WT	c-PTC	-146 (G>A)
279/280	18	VI	-124 (G>A)	c-PTC	WT
325/326	35	No cells	-	Cystic-PTC	-124 (G>A)
355/356	24	IV	WT	FV-PTC	-124 (G>A)
383/384	14	No cells	-	SV-PTC	-124 (G>A)
413/414	70	II	WT	FV-PTC	-146 (G>A)

TERTp - telomerase reverse transcriptase promoter;NIFT: noninvasive follicular thyroid neoplasm with papillary-like nuclear features; PTC: papillary thyroid carcinoma; FTC: follicular thyroid carcinoma. PTC variants: c-PTC- classical; FV-PTC -follicular; SV- PTC solid; TC-PTC- tall cells; HV- PTC hobnail PTC.

Supplementary Table S7B. Profile of the molecular cyto-histological discordant cases in *BRAF* gene.

BRAF **Case number**	**Tumor size mm**	**Cytology by Bethesda categories**		**Histology**	
		**Diagnosis**	**Molecular status**	**Diagnosis**	**Molecular status**
43/44	13	II	WT	TC-PTC	p.V600E
49/50	12	VI	WT	c-PTC	p.V600E
73/74	15	V	p.V600E	FV-PTC	WT
79/80	22	V	WT	FV-PTC	p.V600E
83/84	15	V	WT	c-PTC	p.V600E
93/94	25	V	WT	HV-PTC	p.V600E
101/102	12	IV	WT	FV-PTC/c-PTC	p.V600E
119/120	20	II	WT	c-PTC	p.V600E
129/130	20	VI	WT	FV-PTC	p.V600E
157/158	20	I	WT	c-PTC	p.V600E
173/174	25	VI	p.V600E	c-PTC	WT
197/198	15	III	WT	FV-PTC	p.V600E
201/202	16	III	WT	FV-PTC	p.V600E
221/222	16	V	WT	c-PTC	p.V600E
251/252	25	I	WT	c-PTC	p.V600E
319/320	20	III	WT	HV-PTC	p.V600E
321/322	17	I	NO CELLS	c-PTC	p.V600E
359/360	26	IV	WT	c-PTC	p.V600E
373/374	15	II	WT	FV-PTC	p.K601E
401/402	20	VI	p.V600E	c-PTC	WT
407/408	28	VI	WT	FV-PTC/c-PTC	p.V600E

B-Raf proto-oncogene, serine/threonine kinase; NIFT: noninvasive follicular thyroid neoplasm with papillary-like nuclear features; PTC: papillary thyroid carcinoma; FTC: follicular thyroid carcinoma. PTC variants: c-PTC- classical; FV-PTC -follicular; SV- PTC solid; TC-PTC- tall cells; HV- PTC hobnail PTC.

Supplementary Table S7C. Profile of the molecular cyto-histological discordant cases in *RAS* gene.

NRAS **Case number**	**Tumor size mm**	**Cytology by Bethesda categories**		**Histology**	
		**Diagnosis**	**Molecular status**	**Diagnosis**	**Molecular status**
121/122	45	III	WT	FV-PTC	p.Q61R
153/154	50	VI	WT	FV-PTC/c-PTC	p.Q61R
167/168	25	V	WT	FV-PTC	p.Q61R
237/238	20	V	WT	FTC	p.Q61R
279/280	18	VI	p.Q61R	c-PTC	WT
325/326	35	III	WT	cystic-PTC	p.Q61R
345/346	37	II	WT	FV-PTC	p.Q61R
347/348	70	I	WT	SV-PTC	p.Q61R
385/386	40	IV	p.Q61R	FV-PTC	WT
421/422	18	IV	WT	FV-PTC	p.Q61R
173/174	17	VI	WT	c-PTC	p.Q61R
443/444	18	III	WT	FV-PTC	p.Q61R
477/478	30	II	WT	Benign	p.Q61R

HRAS Case number	Tumor size mm	Cytology by Bethesda categories		Histology	
		Diagnosis	Molecular status	Diagnosis	Molecular status
27/28	20	VI	p.Q61K	c-PTC	WT
59/60	15	IV	WT	FV-PTC	p.Q61R
137/138	25	I	-	FV-PTC	p.Q61R
203/204	13	III	WT	FV-PTC	p.Q61R
223/224	15	IV	WT	FV-PTC	p.Q61R
229/230	30	III	WT	FV-PTC	p.Q61R
277/278	15	IV	WT	FV-PTC/c-PTC	p.Q61R
313/314	30	III	WT	FC-PTC	p.Q61R
331/332	20	VI	WT	HV-PTC	p.G13D
***KRAS* Case number**	**Tumor size mm**	**Cytology by Bethesda categories**		**Histology**	
		**Diagnosis**	**Molecular status**	**Diagnosis**	**Molecular status**
111/112	15	I	WT	FV-PTC	p.Q61R
113/114	30	I	WT	FV-PTC	p.Q61R
123/124	40	IV	WT	c-PTC	p.Q61R
203/204	13	III	WT	FV-PTC	p.G12R
211/212	15	I	WT	NIFT	p.Q61R
283/284	22	V	p.Q61R	c-PTC	WT
343/344	35	III	p.Q61R	FV-PTC	WT
413/414	70	II	p.Q61R	FV-PTC	WT
445/446	30	III	WT	BENIGN	p.G12A

Legend: ND: Non-diagnostic, B: Benign, AUS: Atypia of Undetermined Significance, FN: Follicular Neoplasm, SM: Suspicious for malignancy and M: Malignant. WT: Wild-Type.; *NRAS* - NRAS proto-oncogene, GTPase; *HRAS* - HRas proto-oncogene, GTPase, and *KRAS* - KRAS proto-oncogene, GTPase. NIFT: noninvasive follicular thyroid neoplasm with papillary-like nuclear features; PTC: papillary thyroid carcinoma; FTC: follicular thyroid carcinoma. PTC variants: c-PTC- classical; FV-PTC - follicular; SV- PTC solid; TC-PTC- tall cells; HV- PTC hobnail PTC.
